# Decoding the Role of Satellite DNA in Genome Architecture and Plasticity—An Evolutionary and Clinical Affair

**DOI:** 10.3390/genes11010072

**Published:** 2020-01-09

**Authors:** Sandra Louzada, Mariana Lopes, Daniela Ferreira, Filomena Adega, Ana Escudeiro, Margarida Gama-Carvalho, Raquel Chaves

**Affiliations:** 1 Laboratory of Cytogenomics and Animal Genomics (CAG), Department of Genetics and Biotechnology (DGB), University of Trás-os-Montes and Alto Douro (UTAD), 5000-801 Vila Real, Portugal; slouzada@utad.pt (S.L.); lopesfmariana@gmail.com (M.L.); daniela_p_ferreira@hotmail.com (D.F.); filadega@utad.pt (F.A.); anac.escudeiro@gmail.com (A.E.); 2 Biosystems and Integrative Sciences Institute (BioISI), Faculty of Sciences, University of Lisboa, 1749-016 Lisbon, Portugal; mhcarvalho@fc.ul.pt

**Keywords:** satellite DNA, genome architecture, chromosome restructuring, Robertsonian translocations, satellite DNA transcription

## Abstract

Repetitive DNA is a major organizational component of eukaryotic genomes, being intrinsically related with their architecture and evolution. Tandemly repeated satellite DNAs (satDNAs) can be found clustered in specific heterochromatin-rich chromosomal regions, building vital structures like functional centromeres and also dispersed within euchromatin. Interestingly, despite their association to critical chromosomal structures, satDNAs are widely variable among species due to their high turnover rates. This dynamic behavior has been associated with genome plasticity and chromosome rearrangements, leading to the reshaping of genomes. Here we present the current knowledge regarding satDNAs in the light of new genomic technologies, and the challenges in the study of these sequences. Furthermore, we discuss how these sequences, together with other repeats, influence genome architecture, impacting its evolution and association with disease.

## 1. Introduction

The linear organization of DNA sequences in the genome and how these sequences are packed into chromosomes define their architecture and influence its evolution. Repetitive DNA represents a major organizational component of eukaryotic genomes and includes sequences dispersed throughout the genome like transposable elements (TEs) and tandemly repeated sequences, such as satellite DNA (satDNA) [1,2]. Together with TEs, satDNAs contribute significantly to the differences in genome size between species, accounting for more than 50% of some species total DNA [3]. SatDNAs can be found in varied locations in the chromosomes, such as pericentromeric, subtelomeric and interstitial regions, forming blocks of constitutive heterochromatin (CH) [2,3,4,5,6,7] that are part of vital structures like centromeres and telomeres [2]. However, satDNA location is not restricted to CH with some satDNAs being found also dispersed throughout euchromatic regions in different species [5,8]. Multiple lines of evidence show that satDNAs have key roles in centromere function, heterochromatin formation and maintenance and chromosome pairing [9,10,11,12]. Interestingly, despite their association to critical chromosomal structures, satDNA families can display an astounding sequence variation even among closely related species. This results from their highly dynamic behavior, leading to rapid changes in sequence composition and array size within short evolutionary periods, which can lead to speciation (reviewed in [13]). Moreover, these sequences have been consistently correlated with fragile sites and evolutionary breakpoint regions in diverse species [14,15,16,17,18,19] and are intrinsically involved in frequent chromosomal rearrangements like Robertsonian translocations [20,21]. SatDNA dynamics has been shown to promote genome plasticity and to have an active involvement in the modulation of genomic architecture by promoting rearrangements.

Nevertheless, some satDNAs seem to have been preserved or “frozen” across different taxa during long evolutionary periods [22,23,24] with some of them being transcribed into satellite non-coding RNAs (satncRNAs). Indeed, transcripts of satDNAs have been reported in different species, highlighting a possible role for satncRNAs in the regulation of gene expression, cancer outcomes and aging [25,26,27]. This suggests that functional constraints may be causing the preservation of these sequences over the time [24,28]. Accordingly, some species centromeric satDNAs have been found to share a 17 bp motif known as the centromere protein B (CENP-B) box, representing the binding site for centromere protein B (CENP-B) [29,30]. It has been demonstrated that the CENP-B box is required for de novo centromere chromatin assembly and CENP-B protein is involved in centromere functions [31]. In this case, the conservation of a sequence motif across diverse mammalian species satDNAs [32] seems to be related to a specific function.

Over the years, different techniques have been used to address satDNA sequences. The advances in sequencing technology and computational approaches have revolutionized the study of these regions, known as the “black holes” of the genome. The increasing number of studies assessing the genomic abundance and sequence variation of satDNAs in different species has led to the coining of new terms to describe the whole collection of repeats (repeatome) and satDNAs (satellitome) in a species genome [33,34], and contributed to improve our knowledge regarding the evolution and function of these sequences [35].

In this review, we contextualize satDNA sequences in the genomes/chromosomes of different species in the light of recent data provided by new technologies and bioinformatic tools and the challenges of studying these DNA sequences and their associated non-coding RNAs. We also discuss the contribution of repetitive sequences to the organization of genomes and their participation in the restructuring of species karyotypes during evolution, focusing on their involvement in rearrangements with evolutionary and clinical significance: Robertsonian translocations. Finally, we address the structural role of satDNA transcripts in the genome.

## 2. SatDNA Features and Organization in the Genome and Chromosomes: Emerging Technologies and Changing Concepts

The concept of satDNA suffered considerable changes through time. Early experiments historically coined the term “satellite DNA” referring to tandemly arranged sequences that formed satellite bands separate from the rest of the genomic DNA during density gradient centrifugation [36]. Given that no function was initially attributed to these sequences, they were considered as genomic “junk”, representing parasites proliferating independently in the genomes [37]. Today, satDNAs are viewed as important genomic functional components. In order to understand participation of these sequences in genome architecture and evolution, we need to briefly address their organizational features, localization and mode of evolution.

SatDNA is typically organized as long arrays of head-to-tail linked repeats and usually present in the genomes in several million copies [1]. The length of the repeating unit (monomer) can range from a few base pairs up to more than 1 kb, forming arrays that may reach 100 Mb in length (reviewed by [38]), and that can form higher-order repeat (HOR) units (e.g., [39,40,41]). Human chromosome centromeres are populated by α satDNA (*αSAT*) organized in HORs that are structurally distinct and confer chromosome specificity [39,42]. Complex HORs have been found in non-human mammals such as insects, mouse, swine, bovids, horse, dog and elephant (reviewed in [43]), and more recently in Callitrichini monkeys [44] and Teleostei fish [45]. SatDNA arrays are mainly found clustered in heterochromatin, although studies also report the presence of short satDNA arrays dispersed along euchromatic regions [2,3,4,5,6,7]. These sequences can be found in varied locations in the chromosomes, such as pericentromeric, subtelomeric and interstitial regions [2,46,47,48], as well as being part of vital structures like centromeres and telomeres [2].

Usually more than one family of satDNAs can be found in the same genome, thus forming a library, which can be shared among closely related species. The satDNAs within the library may differ in monomer sequence, size, abundance, distribution and location (reviewed in [12]). Expansions and contractions of satDNA arrays can dramatically change the landscape of repetitive sequences, leading to significant differences of satDNA copy number among related species [49,50]. That is the case of the *Drosophila* genus, which contains very dissimilar satDNAs, varying from 0.5% in some species genomes to as high as 50% in others [51,52]. Such striking differences in satDNA abundance in *Drosophila* sp. were proposed to result predominantly from lineage-specific gains accumulated over the past 40 MY of evolution [53], ultimately causing species reproductive barriers [54,55].

The mechanisms proposed to be responsible for the amplification/deletion of repetitive DNA, consequently leading to their rapid evolutionary turnover, are unequal crossing over, replication slippage and rolling circle amplification [56]. SatDNA sequence divergence among species is quite variable, as some repeats are species-specific, while others are widely conserved, being shared across distantly related species [22,24,57]. SatDNAs have a unique mode of evolution, known as concerted evolution, a two-level process in which mutations are homogenized throughout monomers of a repetitive family and concomitantly fixed within a group of reproductively linked organisms [58,59].

The study and characterization of satDNA has lagged behind when compared with other genomic sequences. Throughout time, different methodological approaches have generated insights into the structure, organization, function and evolution of these sequence elements, although this characterization has been significantly hampered by their highly repetitive nature. The advent of high-throughput sequencing technologies and associated bioinformatics tools opened the door to whole genome sequencing projects, and as the technology became more robust and cheaper, the number of sequenced species increased exponentially. In 2018, the Earth BioGenome project was launched, aiming to increase the number of sequenced eukaryotic genomes from 2534 species (of which only 25 comply with the standard for contig and scaffold N50 established by the Genome 10K organization) to characterize the genomes of the 1.5 million known species within a 10 year time frame [60]. Of note, satDNA, as well as other repetitive sequences, have been systematically omitted from the genome projects, due to difficulties in sequence alignment and assembly, given that the read length of current sequencing technologies is unable to span the longer repeats and tandem arrays [61,62]. Nevertheless, high-throughput sequencing contributed significantly to increase our knowledge regarding satDNA sequences [63]. Next generation sequencing (NGS; e.g., Illumina), allied to newly developed bioinformatics tools capable of identifying satDNA sequences in unassembled data (e.g., RepeatExplorer) [64,65,66], helped uncover the extent of satDNAs present in the genome of different species, revealing unpredicted levels of satDNA diversity (e.g., [34,67,68,69,70,71]). For instances, 62 satDNA families were identified in the genome of the migratory locust, leading to the coining of the term ‘satellitome’ to refer to the whole collection of satDNA families found in a single genome [34], a part of the ‘repeatome’, a term proposed previously [33] to refer to the collection of all repetitive sequences in a genome (TEs, satDNAs, etc.). This number has been surpassed by a recent study where 164 satDNA families have been identified in Teleostei fish, being this the biggest satellitome characterized for a given species so far [70]. The availability of a methodology capable of assessing satDNA array abundance and diversity led to an explosion of comparative studies across a wide range of clades, including mammals, insects and plants (e.g., [44,45,69,71,72,73]) providing insights into these sequences.

The development of sequencing technologies that generate long-range data has allowed the community to overcome some of the limitations imposed by NGS and is fueling the study of repeats. Single-molecule real-time sequencing and nanopore sequencing technologies (commercialized by PacBio and Oxford Nanopore Technologies (ONT), respectively) can generate longer reads capable of spanning repetitive regions, thus enabling their assembly into contigs (reviewed in [62]). For instances, ONT nanopore sequencers have been shown to generate unprecedented ultra-long reads that can reach mega-base lengths, leading to significant improvements in the human genome assembly [74,75,76,77], with some of the repetitive-containing gaps being closed [78,79]. By using long-read methods we are gaining access to important repeated-rich structures, like centromeres, revealing further insights into their sequence content and structure [80]. For instances, *Drosophila* centromeric satDNAs were recently shown to be intermingled with TEs [81]. Other recent studies report the improvement of human Y chromosome centromere assembly [78] and the reconstruction of a 2.8 megabase centromeric satDNA array, with the potential to achieve for the first-time telomere-to-telomere sequencing of the X chromosome [79].

Several studies demonstrate that the combination of different high-throughput sequencing methods (e.g., Illumina, ONT and PacBio) with other techniques, such as optical mapping, cytogenetics and molecular techniques, is beneficial and sometimes essential to determine satDNA features. The use of PacBio long-read sequencing together with optical mapping proved to be helpful in the assembly of satDNA arrays with large monomers and provided insights regarding recombination rates in the Eurasian crow [82]. Positional data derived from fluorescent in situ hybridization (FISH) remains vital to determine the physical location of satDNAs, since such information cannot be achieved for genomes that have not yet been properly assembled (e.g., [34,44,71,81,83]), and sequences mapping by FISH on extended DNA fibers can provide significant assistance to the process of genome assembly, aiding in contig ordering (e.g., [84,85]). Improved techniques based on FISH, helped shedding light into repetitive-rich chromosome regions with centromeric function (e.g., [86]). Other methods have also shown to provide a valid and expedite analysis of repetitive sequences profile, such as PCR-based approaches, that have been used to determine satDNA copy number differences between healthy and cancer cells/tissues [87]. In particular, the use of droplet digital PCR (ddPCR) combined with other methodologies has contributed to the validation and quantification of rare retrotransposon insertion events in different tissues including tumors [88] and the detection and accurate quantification of human *SATII* ncRNA in cancer patients [89]. The integration of genomic, cytogenetic and cell biology data helps to establish a connection between sequence information, its localization in the chromosomes and their interaction with other components of the genome, defining the field of chromosomics [90]. We believe that this approach is essential to fully understand the organization of repetitive sequences.

Other aspects of satDNA biology are also becoming accessible through the use of recent methodologies, such as the characterization of their expression and chromatin state, namely by using RNA sequencing (RNA-seq) and chromatin immunoprecipitation approaches followed by DNA sequencing (CHIP-seq) [91,92]. In particular, for CHIP-seq experiments several studies report the use of a specific antibody against DNA binding centromere-specific histone H3 (CENH3), which is an ortholog for human CENP-A. This methodology has proven to be useful for clarifying the satDNA content in the centromere, improving some organisms reference sequence and uncovering satDNA variability (e.g., [93,94]). 

**Figure 1:**
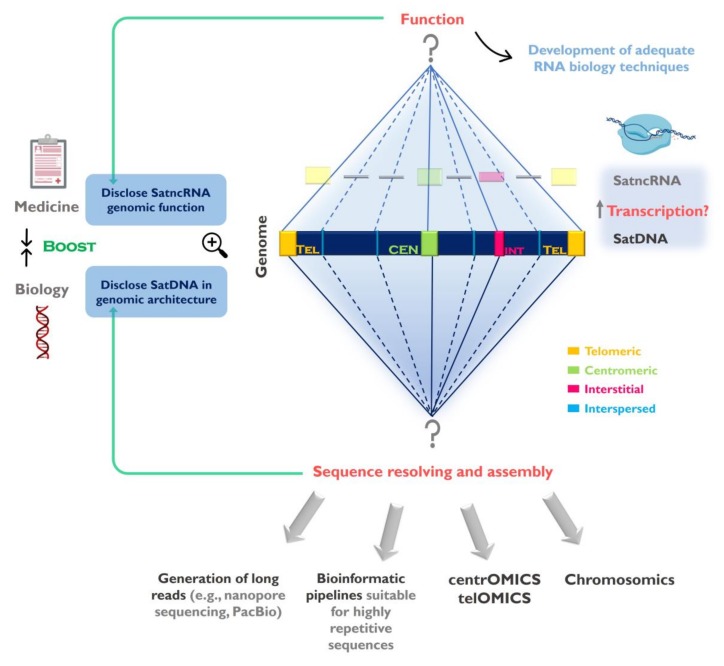
Challenges in the study of satellite DNA (satDNA) sequences and the importance to fully understand the repetitive genomic fraction. SatDNAs can be found clustered at the centromeres, telomeres and forming interstitial heterochromatin (CH) blocks, as well as scattered (interspersed) throughout the chromosomes. The full characterization of satDNAs needs to be addressed in two levels: 1-Disclose satDNAs linear sequence and improve their representation in genome assemblies. Despite currently used sequencing strategies (e.g., next generation sequencing (NGS)) contributed for satDNA studies, the full characterization of these sequences will only be achieved by using sequencing technologies capable of long reads, bioinformatics pipelines suitable for highly repetitive sequences, together with other techniques (e.g., FISH, optical mapping). These strategies need to be directed to specific chromosome structures such as centromeres (centrOMICs) and telomeres (telOMICs), which harbor large amounts of satDNA. Important also is the integration of genomic data with sequence localization in the chromosomes, and their interaction with other components of the genome (chromosomics); 2- Clarify satDNAs function(s) in the genome by studying the satellite non-coding RNAs (satncRNA) and their interaction with other components and structures in the genome. In this field there is the need to develop adequate biology techniques to address repetitive sequences transcription study. The disclosure of satDNA sequences will help to better understand its genomic architecture ant its role in genome restructuring in evolution and disease.

The data generated is now being used to determine satDNA sequences organization in the genome [95], explore predicted evolutionary patterns and hypothesis (e.g., [35,68,96,97]), as well as to shed light into the function of these sequences [81,98]. We are now closer than ever to fully access the sequence information hidden within repetitive-rich chromosome structures like centromeres and telomeres. However, we still need to further develop and adapt currently available approaches to achieve a combination of genomic, cytogenetic and molecular techniques to optimally address these regions, which we propose could be referred to as centrOMICs and telOMICs (Figure 1). SatDNAs represent one of the most intriguing and also interesting components of the genome and their full characterization will help us to better understand genome organization, architecture and evolution.

## 3. Modulating Genome Architecture with SatDNAs

The architecture of genomes confers identity to species. From a generalist point of view, the genomic architectural configuration is the product of a series of sequential molecular events that occurred during the evolutionary process. The impact of these events on genome organization is reflected by chromosome size, number and morphology. Eukaryotic genomes, and particularly, karyotypes, can be viewed as a set of homologous chromosomes, each harboring a combination of syntenic blocks—conserved blocks that can be differently assembled between species [99]. The events with capability for shaping genomes are based on structural and quantitative chromosomal alterations (e.g., [100]) of variable dimensions, from small to large regions that may completely change the morphology and number of species chromosomes and karyotypes. Amongst these, chromosome fusions (i.e., Robertsonian translocations), fissions (reviewed in [99]) and inversions [101], are perhaps the ones with a stronger impact on the architectural appearance of genomes during species evolution.

Chromosome structural variation may originate from illegitimate non-homologous recombination between different chromosome fields, such as centromeres, chromosome arms and telomeres during meiosis, requiring double strand breaks in at least two chromosomes or chromosome regions [102,103,104,105]. The resulting rearranged chromosomes are transmitted either as potentially harmful alterations, or as new variants associated with a selective advantage that will eventually conduct to speciation [99,106,107].

Even before the routine use of advanced molecular technologies, cytogeneticists could realize that the regions where chromosomes break and rearrange (the so-called chromosomal breakpoints) were enriched in constitutive heterochromatin, evidenced by C-bands [57]. Molecular technologies demonstrated that evolutionary breakpoint regions are composed of repeats [107,108,109,110,111]. The involvement of repetitive sequences, including TEs (e.g., [112,113,114,115]), segmental duplications (e.g., [108,110,116]) and tandem repeats (e.g., [14,15,16,17]), in genome restructuring and evolution is now widely recognized. 

The evolutionary rate of tandemly repeated satDNA was shown to be higher than in other genomic sequences, presenting significant changes in short evolutionary times. It is thought that the mechanisms leading to the rapid turnover of these sequences promote chromosome rearrangements and consequently contribute to re-shaping of the genomes. Unequal crossing-over events seem to be responsible for the rapid evolution and divergence found among satDNA families, specifically at the levels of monomer length, nucleotide sequence, complexity and copy number [1,14,49,117,118]. DNA polymerase slippage during DNA replication and recombination in meiosis caused by faulty alignment of repetitive elements further contributes to the instability of these repeat rich regions and to chromosome rearrangements (e.g., [107,119,120]). 

SatDNAs can display complex structural organization resulting from the formation of secondary DNA structures, including hairpins, triplexes [121] and even tetraplexes (G-quadruplexes) [122,123]. The formation of such structures can cause problems during genome duplication in the S-phase by slowing down or even stalling the replication fork, resulting in double-strand breaks [124,125]. This damage is then targeted for repair by means of homologous recombination-based mechanisms, which may lead to chromosome and genome architecture alterations due to the selection of identical sequences in non-homologous regions as the template for repair [1,19,126].

Several studies document the presence of TEs intermingled with centromeric satDNA [127,128], in some cases forming complex structures [24,129,130,131]. TEs are highly represented in some vertebrate species, making up to 60% or more of their genomes. They are characterized by their mobility within genomes using either a direct cut-and-paste mechanism to alter their position (transposons) or requiring an RNA intermediate (retrotransposons) [127,132]. This intrinsic feature makes them active elements of the genome and has been associated with genomic instability. TEs may cause double strand breaks, not only during the transposition process itself, but also by TE–TE ectopic recombination, which may lead to chromosomal rearrangements and consequently to alterations in the genome architecture [133,134,135,136]. The integration of TEs in the genome may also result in the disruption of a functional DNA sequence (reviewed in [128]), which can have adverse consequences. Together with segmental duplications, these elements share a high degree of similarity between different intra- and inter-chromosomal regions, making them the perfect templates for non-allelic homologous recombination [137,138,139]. TEs dynamics has shown to be linked with satDNA origin and evolution. Evidences suggest that some mobile elements may lead to the generation of new repetitive sequences that can be amplified into long arrays of satDNAs [140]. Moreover, it has been suggested that the autonomous LINE-1 retrotransposons could enable amplification and intragenomic movements of satDNA sequences throughout the genome [141]. It thus seems plausible to think that TEs, especially retrotransposons may, in fact be an adjuvant for satDNA evolution and consequently lead to the creation of genomic innovations.

The dynamic nature of repetitive elements is clearly a basilar reason for genomic plasticity (e.g., [14,102,142,143]) and it is in fact a way of having a low impact on the euchromatic genome [14,97]. Today, an increasing body of evidence strongly validates the involvement of satDNA in the modulation of genomic architectures of a large number of taxa, as in the case of bovids [21,104,144,145], rodents [17,111,143,146], suiformes [57] or genets [147]. This largely extends beyond mammalian evolution, as it can also be observed in insects (e.g., [54,148]), reptiles (e.g., [149]), plants [150] and many other lineages. SatDNAs and TEs can thus be considered the ‘engine’ triggering genome evolution [14,107], with the regions harboring these sequences functioning as ‘hotspots’ or fragile sites for structural chromosome rearrangements, leading to species-specific genome architectures [14,17,57,107,139,143,151] and contributing to the generation of key variations responsible for the success of vertebrates [152].

### 3.1. Repetitive Sequences, Chromosome Instability and Disease

Alterations of genomic architecture can also be pathogenic and have a detrimental effect in organisms, either if occurring at the germinal lineage or somatically. This is the case of many diseases caused or boosted by genomic instability that impacts on nuclear architecture, such as cancer, neurodegenerative disorders and other genetic diseases [153,154,155]. In fact, alterations in genome architecture can interfere both with chromosomal territories and with topological positioning of chromosomes and genes in the nucleus. Due to the constraints in the regulation of genes and gene networks and to differences in somatic mutation frequencies between genome regions located at the nuclear periphery or core (higher in the periphery) [156], structural variations of critical genome regions can in fact threaten normal cell function. Again, in these situations, repeats seem to be the main actors at play [125,128,153,157]. The repetitive fraction of the eukaryotic genome and in particular, of the mammalian genome, is usually methylated and repressed by a highly condensed chromatin state, which seems to be essential to maintain genome integrity (reviewed in [158,159]). When perturbation of the epigenetic landscape of specific genomic regions occurs (e.g., [160]), repeats that are usually silenced can become active and unconstrained, which may lead to mobilization (in the case of TEs) and an open chromatin state that allows the occurrence of double strand breaks at fragile or hotspot regions. This results in chromosome rearrangements with impact on the three-dimensional genome architecture and gene expression regulation [100], which may lead to disease onset and progression. 

#### 3.1.1. Remodeling Genome Architecture Through Robertsonian Translocations from a SatDNA Perspective

The most frequent rearrangements occurring in genomes are Robertsonian translocations (rob). These rearrangements are commonly found in two different genomic scenarios: as an evolutionary rearrangement involved in mammalian karyotypic evolution; and as a chromosomal abnormality with clinical/polymorphic meaning [161,162]. The occurrence of Robertsonian translocations involves a break near or at the centromeric region, followed by the fusion of the entire long (q) arms of two acrocentric chromosomes, forming a dicentric or monocentric chromosome. The associated breakpoints, as well as the subsequent mechanistic steps, have been shown to involve reorganization of satDNA sequences at the centromere level [14,20]. The illegitimate recombination between homologous sequences, such as satDNA on non-homologous chromosomes, has been suggested as a possible path for the occurrence of Robertsonian translocations in mice and humans [163,164]. In fact, the high frequency of rob chromosomes linked to genome remodeling events can be caused not only by the homology of the satDNA sequences shared by the acrocentric chromosomes involved in each translocation, but also by the nicking activity of the centromere protein B (CENP-B), originating the double-strand breaks that precede the fusion events [165]. Robertsonian translocations are complex rearrangements that require, in addition to the double-strand breaks, mechanisms of repair, the silencing of possible additional centromeric sequences and the adjustment of the amount of CH/satDNA over time, in order to maintain chromosome viability [20,162]. This assigns a primordial task to satDNA in the control, success and viability of Robertsonian translocation events [14,162].

One of the well-known examples of the dual character of these rearrangements is the rob (1;29), which assumes a special relevance as it is the most widespread chromosome rearrangement occurring in domestic cattle with clinical significance [166,167,168,169]. In parallel, the rob (1;29) is also a constitutional chromosome rearrangement fixed in several wild bovid species, such as most of the Tragelaphini [170].

The analyses of the sequences at the breakpoint regions preceding a translocation are of great importance in understanding the translocation event [21,131]. These sequences are essentially centromeric satDNAs, whose detailed physical and organizational analysis contributed much to better comprehend the chromosomal mechanism behind the rob (1;29) translocation [20,145]. In 2000, Chaves and colleagues suggested that this chromosomal abnormality might not be a single event [144] and in 2003, using centromeric satDNA sequences, the same group proposed, for the first time, a two-step mechanism for this rearrangement [20]. This translocation mechanism involved, besides the centric fusion of the two acrocentric chromosomes, the loss and reorganization of specific satDNA families that were retained in the translocated chromosome [20]. Later, Di Meo and colleagues [145], using both satDNA and BAC probes, validated the pericentric inversion previously proposed [20]. This event would probably be necessary for satDNA reorganization at the centromeric level, highlighting the active role of satDNA sequences in the translocation mechanism and reinforcing their functional relevance in genome reorganization [14,20].

In humans, the Robertsonian translocations are also the most common structural chromosome abnormality [171,172], with rob (13;14) and rob(14;21) being the most frequent examples [163]. During several decades, aspects such as the high frequency of de novo robs in the human population, their origin during oogenesis, and the non-random participation of the acrocentric chromosomes, have supported the hypothesis that there must be a specific mechanism leading to the formation of these robs [163,164,173]. However, and despite the high frequency of these rearrangements and their clinical implications, there is still insufficient information on the molecular mechanism and exact genomic location of the breakpoints [174]. The rob translocation event has been deeply connected with satDNA sequence homology and consequent recombination [174] giving rise to two alternative explanations: (i) the presence of a homologous inversely-oriented segment on chromosome 14 shared with chromosomes 13 and 21 [163,175]; (ii) the human satellite DNA *SATIII* ability to form uncommon DNA structures that could facilitate the illegitimate recombination [176,177]. However, these hypotheses need further research to be validated. Indeed, in the study of Robertsonian translocations, finding the breakpoint location is a problematic task due to the low resolution of the physical maps at the centromere and short arms of the acrocentric chromosomes [174]. Highly repetitive satDNA undoubtedly represents a major gap in the current human genome assemblies, significantly contributing to the lack of high-resolution sequencing studies in the field of centromere genomics [74,178].

## 4. Transcribing SatDNAs: Targeting Genomic Functions

**Figure 2:**
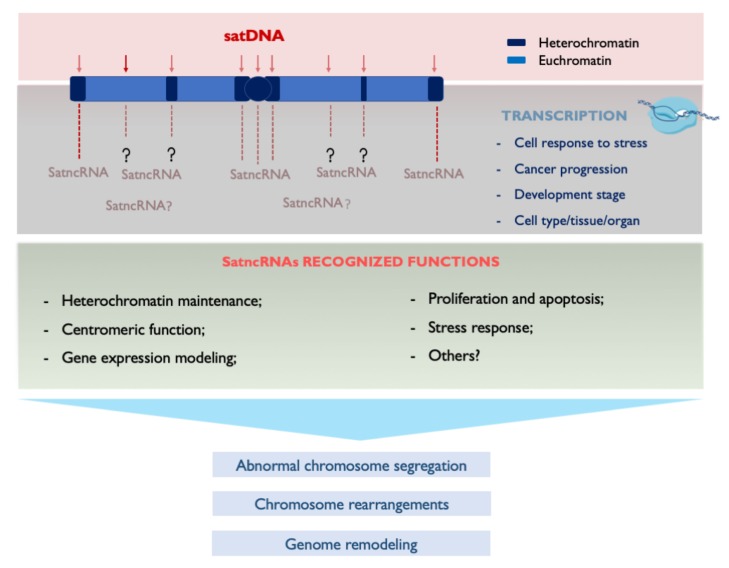
Summary of current knowledge regarding satellite non-coding RNAs (satncRNAs) and how they can contribute to genome remodeling. Even though satDNAs present in the heterochromatin and euchromatin can be transcribed, the most studied satncRNAs are the ones originated from pericentromeric and centromeric satDNAs families. For some satncRNAs reported, chromosome location of the origin satDNA cannot be determined. SatDNA transcription has been shown to be associated to cells response to stress, cancer progression, particular developmental stage and some are differentially expressed in specific cell types, tissues and organs. General recognized functions attributed to satncRNAs are listed. The aberrant expression of satncRNAs may result in abnormal chromosome segregation, and chromosome rearrangements that re-shape the genome and can lead to cancer progression or be fixed during species evolution. Further effort is needed to identify and better characterize satncRNA and their involvement in cellular functions and disease.

The previous sections highlight the role of satDNA sequences in genome architecture and in specific chromosomal rearrangements. However, the participation of these sequences in shaping genome architecture goes beyond their DNA molecule. Currently the transcription of satDNA is a widely accepted feature across species. Different functions have been assigned to satellite non-coding RNAs (satncRNAs) in several cellular contexts, such as cell proliferation, stress response, development or cancer [27] (Figure 2). In fact, satDNA transcripts seem to participate in the most primordial concept of genomic function, being related to centromere structure, chromosome pairing/segregation and kinetochore assembly [2,27,179]. Moreover, recently satDNA transcripts have shown to be associated with male fertility in *Drosophila* sp. [180]. Unfortunately, the function of most satncRNAs remains unknown or unclear due to the inefficient methodologies currently available to analyze molecules of such repetitive nature. 

Concerning centromeric satDNAs, the human α satellite transcripts (*αSAT*) have been shown to be crucial for cell cycle progression, as depletion of *αSAT* resulted in defective centromeric protein A (CENP-A) loading and cell cycle arrest [181]. *αSAT* ncRNAs also seem to regulate spindle microtubule attachment and sister chromatid disjunction through association with AURORA B proteins [182]. These molecules were further shown to be associated with the SUV39H1 histone methyltransferase, thereby suggesting a regulatory function in heterochromatin maintenance [183,184,185].

Contrary to what is believed for most condensed genomic regions, centromeric sequences remain transcriptionally active during mitosis [186,187], essentially promoting kinetochore stabilization and centromere cohesion [188,189]. These functions have been similarly attributed to transcriptionally active centromeric satDNAs from other species [190,191,192], in spite of the observed sequence differences. This suggests that satncRNAs are involved in critical functions, which appear to be associated with their intrinsic molecular characteristics and most probably also with the genomic location of their satDNA sequence.

Pericentromeric satDNA transcripts have been related with pericentric chromatin formation [193,194,195], acting as molecular scaffolds for the accumulation of HP1 [194]. The presence of human *SATIII* ncRNA can be closely associated with cell response to stress. Particularly, heat shock can trigger *SATIII* transcription by the action of HSF1 (Heat Shock Factor 1) [196,197], giving rise to nuclear stress bodies (nSBs) close to *SATIII* DNA regions [198]. The splicing of relevant genes for stress response may be influenced by *SATIII* ncRNAs, which have been proposed to sequester RNA processing factors and downregulate global transcription [199], providing protection against stress-induced cell death [200]. However, *SATIII* transcription is not thermal stress-exclusive, as a basal level of expression is detectable even in the absence of cellular stress [201]. This same satDNA family can exhibit different genomic locations and its transcripts can be involved in multiple functions, making their study even more difficult. *SatDNA III* from *Drosophila melanogaster* is located at the centromere and pericentromere of the X chromosome and at the pericentromere of chromosomes 2 and 3 [202]. Its transcripts have been shown to play different roles in chromatin silencing/heterochromatinization, centromeric function and upregulation of X-linked genes [191,202,203,204].

Another interesting case is the *FA-SAT*, the major satDNA sequence of *Felis catus* (cat) genome, located at the (sub)telomeres and (peri)centromeres of chromosomes [153] and also in an interspersed fashion [24]. This satDNA is highly conserved in its primary sequence among Bilateria species (e.g., human, *Drosophila*, oyster, cattle, among others), a rare event observed in satDNA sequences [24]. In these species (non-*Felis* species), an interspersed distribution of this satDNA was proposed, with the exception of the other carnivore analyzed, *Genetta genetta*, in which it also presents a centromeric location. Of note, *FA-SAT* is transcribed in all these species [24], and an important conserved function (in cat and human) was ascribed to this ncRNA as a PKM2 interactor involved in the cross-talk between proliferation and apoptosis [205]. In fact, the absence of this satncRNA in both species results in cell death [205]. These transcripts possibly originate from the transcription of *FA-SAT* interspersed DNA (at current knowledge, the common location among these species). A putative connection between *FA-SAT* ncRNAs with cancer was also recently hypothesized [205].

With the progressive acceptance of satDNA transcriptional activity, the aberrant expression of satncRNAs has been increasingly associated with cancer progression (reviewed in [27]). In fact, the observed overexpression of satncRNAs in stress conditions may be comparable to cancer, since loss of sister chromatids cohesion, incorrect chromosome segregation or aneuploidy are common features of both states [206]. Overexpression of satncRNAs in cancer has been reported alongside decondensation and hypomethylation of pericentromeric DNA [154,207]. The transcription of satDNAs, the change in nuclear architecture and the altered sequestration of transcription factors may all be related to gene expression deregulation induced by the hypomethylation of *SATII* and *SATIII* satDNA sequences [208,209]. SatncRNAs may thus be involved in more general disease contexts associated to chromatin decondensation, DNA breaks and subsequent genomic rearrangements [209] (Figure 2). However, the value of satncRNAs as cancer biomarkers is still an unexplored field [27].

Due to their repetitive nature, high copy number and multiple genomic locations (different chromosomes and/or genomic regions), the study of satncRNAs remains a difficult challenge, namely regarding the original genomic location of their DNA sequence and the determination of their primary sequence(s) (Figure 2). Indeed, the most common next-generation sequencing platforms presents significant limitations in the analysis of satncRNA sequences in RNA-seq libraries, namely regarding their inability to assemble large repetitive transcripts from very short reads. This could be overcome in the future with the application of ultra-long read sequencing technology. The fact that most of the methods currently available that are mainly directed towards the analysis of gene coding sequences set a requirement for essential improvements or adjustments in order to support the efficient study of satncRNAs (Figure 1).

Although the importance of satncRNA in normal cell function and disease states is becoming increasingly accepted by the scientific community, in the wake of recent studies on these transcripts, much remains to be understood about their functions in different contexts. This will only be overcome through the development of improved methods for the study of repetitive sequences, as well as the commitment of the scientific community to this field of research.

## 5. Concluding Remarks

In this review we outlined the critical importance of satDNA sequences in driving karyotype evolution and genomic architecture, as well as their involvement in various basic genomic functions. However, to this day, significant technological limitations hinder the progress of this important field in biology and medicine, in particular in the study of diseases involving this genomic fraction. It is imperative to boost the study of satDNA sequences and their transcripts by adapting and developing sequencing technologies and bioinformatics pipelines capable of assembling chromosomes from telomere-to-telomere as well as focused approaches that follow the concept of chromosomics. Significant effort is needed from the entire scientific community to value these important genomic elements, which have been so neglected over time. Only then can we begin to fully understand the largest fraction of our genome.
